# The Effect of White Rice and White Bread as Staple Foods on Gut Microbiota and Host Metabolism

**DOI:** 10.3390/nu10091323

**Published:** 2018-09-18

**Authors:** Fumika Mano, Kaori Ikeda, Erina Joo, Yoshihito Fujita, Shunsuke Yamane, Norio Harada, Nobuya Inagaki

**Affiliations:** Department of Diabetes, Endocrinology and Nutrition, Graduate School of Medicine, Kyoto University, Kyoto 606-8507, Japan; fumanou@kuhp.kyoto-u.ac.jp (F.M.); krikeda@kuhp.kyoto-u.ac.jp (K.I.); erinajoo@kuhp.kyoto-u.ac.jp (E.J.); yfujita9@kuhp.kyoto-u.ac.jp (Y.F.); shyamane@kuhp.kyoto-u.ac.jp (S.Y.); nharada@kuhp.kyoto-u.ac.jp (N.H.)

**Keywords:** Japanese diet, dietary pattern, intestinal biota, prebiotics, rice consumption

## Abstract

The purpose of this study was to examine the influence of two kinds of major Japanese staple foods, white rice and white bread, on gut microbiota against the background in which participants eat common side dishes. Seven healthy subjects completed the dietary intervention with two 1-week test periods with a 1-week wash-out period in cross-over design (UMIN registration UMIN000023142). White bread or white rice and 21 frozen prepared side dishes were consumed during the test periods. At baseline and at the end of each period, fasting blood samples, breath samples, and fecal samples were collected. For fecal samples, 16S rRNA gene sequencing was used to analyze the gut microbiota. After the bread period, the abundance of fecal *Bifidobacterium* genus (19.2 ± 14.5 vs. 6.2 ± 6.6 (%), *p* = 0.03), fasting glucagon-like peptide 1 (GLP-1) (13.6 ± 2.0 vs. 10.5 ± 2.9 (pg/mL), *p* = 0.03), and breath hydrogen (23.4 ± 9.9 vs. 8.2 ± 5.5 (ppm), *p* = 0.02) were significantly higher than those of after the rice period. Plasma SCFAs also tended to be higher after the bread period. White bread contains more dietary fiber than refined short grain rice. These findings suggest that indigestible carbohydrate intake from short grain rice as a staple food may be smaller than that of white bread.

## 1. Introduction

Rice is a traditional staple food of the Japanese diet, but per capita rice consumption in Japan has decreased during the past 50 years [1,2]. Meanwhile, bread consumption in Japan has increased, and rice and bread are now the two major staple foods that supply the main proportion of Japanese energy intake [3].

The dietary pattern of eating rice as a staple food includes lower intake of fat and saturated fat and higher intake of dietary fiber compared with eating wheat flour products as staple foods [4]. A previous cross-sectional study indicates that higher intake of rice and the lower intake of bread are associated with lower prevalence of functional constipation [5]. However, it remains unclear whether this effect is mainly due to the difference of staple foods or dietary constituents including side dishes.

Approximately 10% of the carbohydrates ingested resist pancreatic amylase and escape digestion in the small intestine and remain a main substrate for fermentation in the colon [6]. During the fermentation of these indigestible carbohydrates, the gut microbiota produces short-chain fatty acids (SCFAs) [7,8]. According to some previous studies, SCFAs produced by gut microbiota are associated with lipid metabolism [9] and glucose metabolism in humans [10,11,12].

In our everyday meals, side dishes are different from meal to meal, but staple foods are consumed repeatedly. We hypothesized that dietary intake of indigestible carbohydrates derived from staple foods would have effects on host metabolism via the composition of the gut microbiota. In the current pilot study, we focus on the difference of staple foods and their influence on gut microbiota composition and glucose and lipid metabolism in a two-period crossover design using a commercially available package of side dishes.

## 2. Method

### 2.1. Subjects

Healthy volunteers from our research department (students, technical and research staff) were recruited for this study. Inclusion criteria were the following: (1) those who were not currently taking any medication; (2) those who had no abnormality in physical checkup in the past year. Subjects who had a fever, diarrhea or upper respiratory inflammation during the research period were excluded from analysis. The protocol (UMIN registration UMIN000023142) was approved by Kyoto University Graduate School and Faculty of Medicine, Ethics Committee. The study was conducted at Kyoto University Hospital according to the principles of the Declaration of Helsinki. All subjects gave written informed consent.

### 2.2. Study Design

**Figure 1:**
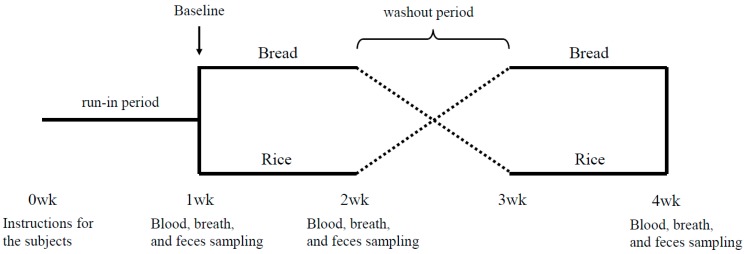
Study design.

The study was a randomized, crossover trial. Following a 1-week run-in period, the subjects were randomized in a 1:1 fashion to one of two intervention sequences: A bread period with supplied side dishes for 1 week followed by a rice period with supplied side dishes for 1 week or a rice period with supplied side dishes for 1 week followed by a bread period with supplied side dishes for 1 week (Figure 1). A 1-week washout period was incorporated between the two test periods. At the baseline and the end of each test period, the blood, breath and fecal samples were collected (Figure 1).

In the run-in and washout periods, the subjects were instructed to avoid eating probiotics, yogurt, oligosaccharides and cultured milk drink. During the test periods, the subjects consumed nothing other than staple food (white bread or white rice) and the supplied side dishes. White bread and white rice on the market were prepared by each subject. The supplied side dishes were a package of the frozen prepared 21 sets of side dishes (TOKATSU FOODS Corporation, Yokohama, Japan). The subjects chose one set of side dishes for each meal in the order they liked during the first 6 days, but on the last day, three sets of side dishes were fixed in both periods. The subjects recorded the amount of bread or rice they ate in the first test period, and they ate the equivalent energy of rice or bread in the second test period. Nutritional content of bread and rice was calculated based on the Food Composition Database published by the Ministry of Education, Culture, Sports, Science and Technology, Japan [13], and nutritional content of side dishes were provided by the manufacturer.

### 2.3. Assessment of Fecal Samples

The fecal samples were collected by subjects at home. The subjects were instructed to put fecal samples in the tubes and put them into boxes with dry ice (−78 °C) immediately after collection, and to bring the boxes to the laboratory. The collected fecal samples were stored at −80 °C until analysis.

16S rRNA gene sequencing analyses of microbial community structure in fecal samples was conducted using a MiSeq (Illumina, San Diego, CA, USA) at TechnoSuruga Laboratory Co., Ltd. (Shizuoka, Japan) according to the method previously described [14]. In brief, PCR amplification was performed by using 341F (5′-CCTACGGGAGGCAGCAG-3′) [15] and 806R (5′-GGACTACHVGGGTWTCTAAT-3′) [16], which were primers for amplifying the V3–V4 region in bacterial 16S rDNA. In addition to the V3–V4 specific priming regions, these primers were complementary to standard Illumina forward and reverse primers. The reverse primer also contained a 6-bp indexing sequence (CAGATC, ACTTGA, GATCAG, TAGCTT, GGCTAC, CTTGTA, ATCACG, CGATGT, TTAGGC and TGACCA) to allow for multiplexing. The touchdown PCR method for thermal cycling was used with a GeneAmp PCR system 9700 (ABI, Foster City, CA, USA). Each PCR reaction mixture (25 μL) contained 20 ng genomic DNA, 2× MightyAmp Buffer Ver.2 (Takara, Otsu, Japan), 0.25 μM of each primer, and 1.25 units of MightyAmp DNA Polymerase (Takara, Otsu, Japan). Each PCR reaction and preparation of amplicon pool were performed as previously described [14].

Each multiplexed library pool was spiked with 12.5% phiX control to improve base calling during sequencing, as recommended by Illumina for the pooling of two libraries [14]. Sequencing was conducted using a paired-end, 2 × 281-bp cycle run on an Illumina MiSeq sequencing system and MiSeq Reagent Kit version 2 (500 Cycle) chemistry. Paired-end sequencing with read lengths of 281 bp was performed. After demultiplexing, a clear overlap in the paired-end reads was observed. This overlap allowed paired reads to be joined together with the fastq-join program (http://code.google.com/p/ea-utils/). The method of quality filtering of sequences was as follows: Only reads that had quality value (QV) scores of ≥20 for more than 99% of the sequence were extracted for further analyses.

Metagenome@KIN software (World Fusion Co., Ltd., Tokyo, Japan) was used to perform homology searching with the determined 16S rDNA sequences, against the TechnoSuruga Lab Microbial Identification Databese DB-BA10.0 (TechnoSuruga Laboratory, Co., Ltd., Tokyo, Japan) which contains only bacteria with standing in the taxonomic nomenclature [17,18]. Bacterial species were identified based on data from 97% similarity cut-off with DB-BA 10.0 [17,18].

### 2.4. Measurement of Blood Samples

The blood samples were drawn after an overnight fast (12 h). At all points, blood samples for measurement of plasma glucose were collected into tubes containing sodium fluoride (NaF) and Ethylenediamine tetraacetic acid (EDTA); blood samples for serum insulin, serum-free fatty acids (FFA) and serum triglyceride (TG) were collected into tubes containing blood coagulation accelerant; and blood samples for incretin were collected into tubes containing dipeptidyl peptidase-4 (DPP-4) inhibitor (BD P800; Becton Dickinson, San Jose, CA, USA). These blood samples were centrifuged (3000 rpm, 20 min, 4 °C), and the collected plasma and serum samples were stored at −80 °C until analysis. Blood samples for short-chain fatty acids (SCFA) were collected into ice-cooled tubes containing EDTA, and were immediately centrifuged (3000 rpm, 10 min, 4 °C). The collected plasma samples were frozen instantly in liquid nitrogen and were stored at −80 °C until analysis.

Plasma glucose was measured by ultraviolet absorption spectrophotometry at SRL, Inc., Tokyo, Japan. Serum insulin was determined using chemiluminescent enzyme immune assay at SRL, Japan. Serum FFA and serum triglyceride were determined using enzymatic colorimetric kits and glycerol-3-phosphate oxidase method, respectively, at SRL, Japan. Total glucagon-like peptide 1 (GLP-1) was measured by human total GLP-1 (ver. 2) assay kit (K150JVC-1; Mesoscale Discovery, Gaithersburg, MD, USA); total glucose-dependent insulinotropic polypeptide (GIP) was measured by human GIP (total) ELISA (EZHGIP-54K; Merck Millipore, Darmstadt, Germany). Plasma SCFA was measured by liquid chromatography coupled with tandem mass spectrometry (LC-MS/MS) at LSI Medience Corporation, Tokyo, Japan.

### 2.5. Analyses of Breath Hydrogen

Endtidal breath samples were collected into aluminum bags at the same occasion as blood sampling in order to measure breath hydrogen, which is an indicator of colonic fermentation [10,11,19,20,21]. Breath hydrogen was measured by simple gas chromatograph (Breath Gas Analyzer BGA1000D) at Laboratory for Expiration Bio-chemistry Nourishment Metabolism Co., Ltd., Nara, Japan [22,23].

### 2.6. Statistical Analysis

The sample size calculation was based on a standardized effect size of 2.5 (breath hydrogen) estimated from a previous study [10]. A sample size of five was needed to provide 80% power to detect this difference at a two-tailed significance level of 0.05.

All data are expressed as mean with standard deviation. Comparisons between samples at the end of bread periods and those at the end of rice periods were performed using paired *t* test. Two-tailed *p* < 0.05 was considered statistically significant. Statistical analyses were performed with JMP version 13 (SAS Institute, Cary, NC, USA).

## 3. Results

### 3.1. Characteristic of Subjects

Ten healthy volunteers participated in this study. Three subjects had a fever or diarrhea in the test period and were excluded from the analysis. Seven healthy subjects (two males and five females; mean (±standard deviation (SD)) age 36.7 ± 4.0 years (range 31–42) and body mass index (BMI) (kg/m^2^) 21.0 ± 1.5 (range 18.6–23.1)) were analyzed. Plasma glucose and serum insulin of all subjects were within normal limits (91.2 ± 2.9 mg/dL, 5.2 ± 1.6 µIU/mL, respectively) (Table 1). Five of the seven subjects (two males and three females; mean (±SD) age 36.2 ± 3.9 years and BMI (kg/m^2^) 20.4 ± 1.3) were analyzed for plasma SCFA, breath hydrogen and intestinal microbiota.

### 3.2. Energy Intake and Dietary Composition

The provided sets of frozen side dishes were composed of three side dishes (one main dish and two small side dishes). Main dishes were made with meat, fish or egg, and two small side dishes were mainly made with vegetables. Further information on the typical Japanese side dishes used in this study can be seen in Supplemental Table S1. The mean energy content of 21 sets of side dishes was 294.7 ± 66.8 (kcal/meal), and the mean energy intake from white bread and white rice were 270.6 ± 43.5 (kcal/meal) and 272.7 ± 32.9 (kcal/meal), respectively (Table 2). In both periods, all subjects consumed the staple foods and supplied side dishes completely. White bread, however, has more protein, fat and fiber, and less carbohydrate compared with the equivalent energy of white rice. The calculated energy composition of protein, carbohydrate and fat were 16.4%, 54.1% and 29.5% in the bread period and 12.8%, 63.8% and 23.5% in the rice period, respectively.

### 3.3. Intestinal Microbiota Composition

An average of 40,754 reads were obtained for each sequencing reaction. The minimum and maximum number of sequencing reads were 35,791 and 46,687, respectively. The abundance was a percentage of each number of read in all sequencing reads.

**Figure 2:**
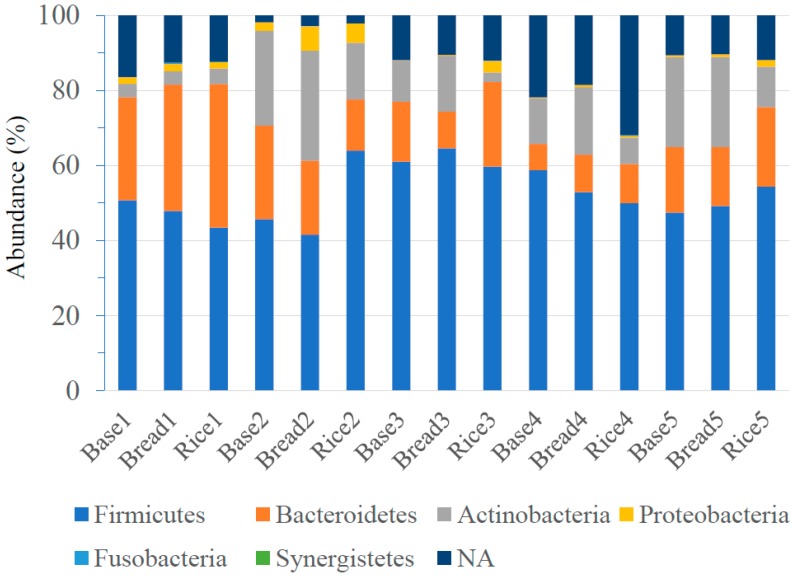
*Phylum*-level classification of bacteria identified in fecal samples of five subjects. The *phyla* represented by the different colors are shown below the figure. Baseline: Base; After bread period: Bread; After rice period: Rice.

**Figure 3:**
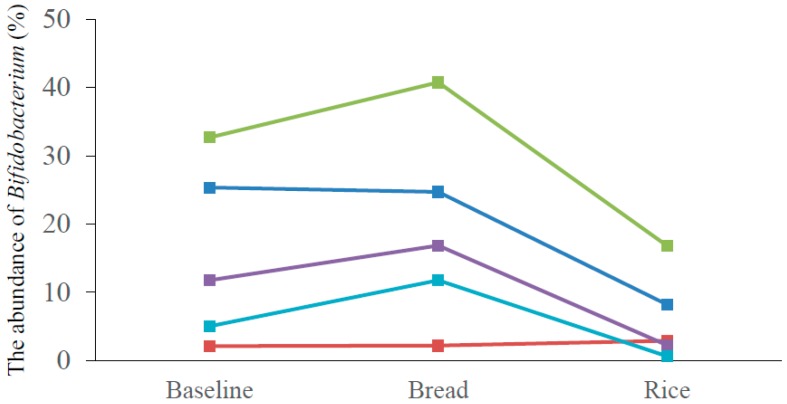
The abundance of *Bifidobacterium* of five subjects at baseline, after bread period and after rice period. After bread period: Bread; After rice period: Rice. Each color indicates each subject.

*Bacteroidetes*, *Firmicutes* and *Actinobacteria* were the major three *phyla*, and dietary interventions did not make any significant difference in the abundance of *Bacteroidetes* and *Firmicutes*. (Figure 2). However, the abundance of *Actinobacteria* was significantly higher after the bread period compared with that after the rice period (18.0 ± 9.8 vs. 7.9 ± 5.1 (%), *p* = 0.02). Class-level analyses revealed that the abundance of *Actinobacteria* was significantly higher after the bread period compared with that after the rice period (18.0 ± 9.8 vs. 7.9 ± 2.3 (%), *p* = 0.02). No significant difference was observed in other classes. The abundance of *Bifidobacteriales* at order-level (14.7 ± 9.9 vs. 5.4 ± 5.5 (%), *p* = 0.02), and the abundance of *Bifidobacteriaceae* at family-level (14.7 ± 9.9 vs. 5.4 ± 5.5 (%), *p* = 0.02) were significantly higher after the bread period than those after the rice period. No significant difference was observed in other orders and families. Genus-level analyses determined that the abundance of *Bifidobacterium* was significantly higher after the bread period than after the rice period (19.2 ± 14.5 vs. 6.2 ± 6.6 (%), *p* = 0.03) (Table 3). Although there was individual difference in the abundance of *Biffidobacterium* at the baseline, four of the five subjects showed higher abundance of *Bifidobacterium* after the bread period compared with those after the rice period (Figure 3). No significant difference was observed in other genera. At species level, the abundance of *Bifidobacterium longum* was significantly higher after the bread period compared with that after the rice period (3.3 ± 3.1 vs. 2.3 ± 3.3 (%), *p* < 0.01). The abundance of *Blautia faecis* was significantly higher after the rice period compared with that after the bread period (0.6 ± 0.4 vs. 1.0 ± 0.7 (%), *p* = 0.046; the abundance at the baseline = 0.8 ± 0.4 (%)). No significant difference was observed in other species.

### 3.4. Hormonal and Metabolic Changes in Blood and Breath

The plasma GLP-1 level after the bread period was significantly higher than that after the rice period (13.6 ± 2.0 vs. 10.5 ± 2.9 (pg/mL), *p* = 0.03) (Table 4). Glucose, insulin, GIP, triglyceride and free fatty acids showed no significant differences. Plasma propionate and butyrate levels tended to be higher after the bread period compared with those after the rice period (0.11 ± 0.09 vs. 0.06 ± 0.03 (µg/mL), *p* = 0.16, 0.06 ± 0.04 vs. 0.02 ± 0.01 (µg/mL), *p* = 0.12, respectively). Breath hydrogen was significantly higher after the bread period than after the rice period (23.4 ± 9.9 vs. 8.2 ± 5.5 (ppm), *p* = 0.02).

## 4. Discussion

In the current study, we examined the influence of staple foods on gut microbiota against the background in which subjects consumed fixed sets of side dishes. Many previous studies on the effect of dietary intervention on gut microbiota were conducted by using foods with a large difference in dietary fiber content [8,10,11,12,20,21] or by adding specific non-digestible carbohydrates to daily meals [24,25,26,27]. The strength of the current study is that the test meals used were very similar to everyday meals of Japanese people; we used two major staple foods, white rice and white bread together with ordinary kinds of side dishes. After the bread period, abundance of fecal *Bifidobacterium*, fasting plasma GLP-1 level, and breath hydrogen were significantly higher than those of after the rice period.Dietary fiber and resistant starch are prebiotics, which act as a fermentation substrate within the colon and stimulate preferential growth and activity of specific microbial species (e.g., *Bifidobacterium*) and confer health benefits on the host [27,28]. The major products of fermentable carbohydrate in gut microbiota are SCFAs (e.g., acetate, propionate, butyrate) and gases (e.g., hydrogen and carbon dioxide) [29]. In this study, 7-days intake of bread containing a higher amount of dietary fiber than rice induced higher abundance of *Bifidobacterium* and higher excretion of hydrogen. At species level, the abundance of *Blautia faecis* which belongs to the genus *Blautia* was significantly higher after the rice period compared with that after the bread period. A previous study reported that the composition of the Japanese gut microbiome showed more abundance in the genus *Bifidobacterium* and *Blautia* compared with those of Western and other Asian people [30]. However, to our knowledge, there is no report that dietary intervention affects the abundance of *Blautia faecis*. 

GLP-1 is an incretin secreted by intestinal endocrine L cells located mainly in the ileum and colon [31]. SCFAs produced by fermentation in gut microbiota can directly enhance L cells’ release of GLP-1 through the SCFA receptors, GPR41 and GPR43 [29,32,33]. GPR41 are activated by propionate and butyrate and GPR43 are activated by acetate and propionate [32,34,35]. In our study, plasma propionate and butyrate levels after the bread period tended to be higher than those after the rice period. These facts support the higher fasting GLP-1 observed after the bread period.

One limitation of this study is that resistant starch content of bread and rice was not measured. Murphy et al. reported in her review article that the mean value of resistant starch content of white bread and white rice was almost the same [36], but in that review, rice included long grain rice cultivars. In Japan, the *japonica* rice cultivars (short grain rice), which contain lesser resistant starch than long grain rice cultivars, are popular [37,38]. In the current study, one possibility is that the resistant starch content of bread was higher than that of rice.

Another limitation of this study is that food records of the subjects’ usual diet before the test periods were not obtained. The baseline values of fecal *Bifidobacterium*, plasma GLP-1, and breath hydrogen were generally higher than those after the rice period. It is possible that the intake of indigestible carbohydrate during the washout period was larger than that during the rice period. The mean amount of dietary fiber consumed in the rice period was calculated to be about 14.4 (g/day), which was comparable to the average amount of the dietary fiber intake of Japanese people, 14.2 (g/day) [3].

Given that the Japanese diet is almost always composed of one staple food and side dishes [2], the choice of staple foods has considerable influence on the intake volume of indigestible carbohydrate. This study suggests that people who consume rice as a staple food (especially short grain rice) may need to consume more dietary fiber from side dishes.

## 5. Conclusions

Against the background of people eating common side dishes, 7 days intake of white bread induced significantly higher abundance of fecal *Bifidobacterium*, fasting GLP-1, and breath hydrogen compared with 7 days intake of white rice.

## Figures and Tables

**Table 1:** Characteristics of the subjects at baseline.

Subjects (*n*)	7
Glucose (mg/dL)	91.2 ± 2.9
Insulin (µIU/mL)	5.2 ± 1.6
GIP (pg/mL)	56.6 ± 31.6
GLP-1 (pg/mL)	15.7 ± 7.5
TG (mg/dL)	64.4 ± 22.3
FFA (µEq/L)	646.3 ± 250.0
Short-chain fatty acids	
Acetate (µg/mL)	6.64 ± 6.14
Propionate (µg/mL)	0.07 ± 0.02
Butyrate (µg/mL)	0.04 ± 0.02
Breath H_2_ (ppm)	15.8 ± 12.0

All values are means ± SD.

**Table 2:** Nutritional composition of bread, rice and side dish per one meal.

	Energy (kcal)	Protein (g)	Carbohydrate (g)	Fat (g)	Fiber (g)
Bread (*n* = 7)	270.6 ± 43.5	9.5 ± 1.5	47.9 ± 7.7	4.5 ± 0.7	2.4 ± 0.4
Rice (*n* = 7)	272.7 ± 32.9	4.1 ± 0.5	60.2 ± 7.3	0.5 ± 0.1	0.5 ± 0.1
Side dish (*n* = 7)	294.7 ± 66.8	13.7 ± 3.2	28.5 ± 7.4	14.0 ± 5.0	4.3 ± 1.0

All values are Means ± SD.

**Table 3:** Mean value of genera of the *Actinobacteria phylum* identified in fecal samples at baseline, and after bread and rice periods.

	Baseline (*n* = 7)	Bread (*n* = 7)	Rice (*n* = 7)
*Bifidobacterium* (%)	15.3 ± 13.2	19.2 ± 14.5 *	6.2 ± 6.6
*Collinsella* (%)	3.2 ± 3.9	3.5 ± 4.5	2.3 ± 2.8
*Eggerthella* (%)	0.3 ± 0.3	0.1 ± 0.1	0.3 ± 0.3
*Actinomyces* (%)	0.1 ± 0.05	0.1 ± 0.1	0.1 ± 0.05

All values are Means ± SD. After bread period: Bread; After rice period: Rice. *p* values are derived by two-tailed paired *t* test. *p* * < 0.05 for Bread versus Rice.

**Table 4:** Plasma or serum concentration of metabolites, and breath hydrogen after bread and rice periods.

	Bread (*n* = 7)	Rice (*n* = 7)	*p* Value
Glucose (mg/dL)	86.2 ± 5.0	87.4 ± 6.9	0.52
Insulin (µIU/mL)	4.0 ± 0.9	3.5 ± 1.1	0.34
GIP (pg/mL)	55.2 ± 18.9	43.8 ± 21.1	0.11
GLP-1 (pg/mL)	13.6 ± 2.0	10.5 ± 2.9	0.03 *
TG (mg/dL)	58.4 ± 16.4	73.9 ± 29.4	0.20
FFA (µEq/L)	619.6 ± 149.1	561.6 ± 281.6	0.63
Short-chain fatty acids			
Acetate (µg/mL)	5.34 ± 4.08	4.12 ± 3.06	0.70
Propionate (µg/mL)	0.11 ± 0.09	0.06 ± 0.03	0.16
Butyrate (µg/mL)	0.06 ± 0.04	0.02 ± 0.01	0.12
Breath H_2_ (ppm)	23.4 ± 9.9	8.2 ± 5.2	0.02 *

All values are means ± SD. After bread period: Bread; After rice period: Rice; *p* values are derived by two-tailed paired *t* test. * *p* < 0.05 for Bread versus Rice.

## Supplementary Materials

The following are available online at http://www.mdpi.com/2072-6643/10/9/1323/s1, Table S1: Menu and food ingredients of the side dish sets on the 7th day of both test periods (bread and rice).
