# Supplementary material for: The Effect of White Rice and White Bread as Staple Foods on Gut Microbiota and Host Metabolism
Source: Nutrients. 2018 Sep 18;10(9):1323. doi: 10.3390/nu10091323 (PMC6164422; doi:10.3390/nu10091323)
Supplement: Supplementary file 1 [file nutrients-10-01323-s001.pdf]

Supplemental table 1 Menu and food ingredients of the side dish sets on the 7th day of both test periods (bread and rice)

		Menu	Food ingredients
Breakfast	Main dish	Chicken meatball and vegetable	Chicken, Chinese cabbage, Mushroom, Noodles made from devil's tongue starch, Green onion, Carrot, Sugar, Soy source, Seasonings (Amino acids, etc.)
	Small side dish 1	*Koya-tofu* (freeze-dried *tofu*) and egg simmered in seasoned broth	Onion, Egg, *Koya-tofu*, Edible clover, Sugar, Soy source, Seasonings (Amino acids, etc.)
	Small side dish 2	Okra and yam with Japanese plum paste	Yam, Okra, Japanese plum paste, shavings of dried bonito, Sugar, Soy source, Seasonings (Amino acids, etc.)
Lunch	Main dish	Omelet with mushroom sauce	Egg, Cheese, Tomato, Mushroom, Onion, Ketchup, Olive oil, Garlic, Sugar, Seasonings (Amino acids, etc.)
	Small side dish 1	Curry flavored sautéed spinach	Spinach, Carrot, Butter flavored oil, salt, Curry powder, Pepper, Seasonings (Amino acids, etc.)
	Small side dish 2	Sweet potato salad	Sweet potato, Almond, Sugar, Honey, Flavors
Dinner	Main dish	Cod fish with Ratatouille sauce	Cod fish, Tomato, Zucchini, White wine, Spanish paprika, Onion, Celery, Carrot, Olive oil, Milk, Salt, Pepper, Garlic, Seasonings (Amino acids, etc.)
	Small side dish 1	Sauteed soybean sprouts and Japanese mustard spinach	Japanese mustard spinach, Soybean sprouts, Carrot, *Miso* paste, Sugar, Soy source, Seasonings (Amino acids, etc.)
	Small side dish 2	Curry flavored boiled potato	Potato, Carrot, Sugar, Pea Honey, Curry powder, Soy source, Salt, Seasonings (Amino acids, etc.)
